# When algorithms infer gender: revisiting computational phenotyping with electronic health records data

**DOI:** 10.1186/s13293-025-00783-8

**Published:** 2025-12-31

**Authors:** Jessica Gronsbell, Hilary Thurston, Lillian Dong, Vanessa Ferguson, Diksha Sen Chaudhury, Braden O’Neill, Katrina S. Sha, Rebecca Bonneville

**Affiliations:** 1 Department of Statistical Sciences, University of Toronto, Toronto, M5G 1X6 ON Canada; 2 Department of Gender, Feminist, and Women’s Studies, York University, Toronto, M3J 1P3 ON Canada; 3 Department of Biostatistics, Dalla Lana School of Public Health, University of Toronto, Toronto, M5S 1A8 ON Canada; 4 School of Health Policy & Management, York University, Toronto, M3N 3A7 ON Canada; 5 Department of Family Medicine, University of British Columbia, V6T 1Z3 Vancouver, BC Canada; 6 Resilient Minds Psychiatry, Ojai, 93023 CA USA

**Keywords:** Computational phenotyping, Bias, Electronic health records, Ethics, Gender, Transgender persons

## Abstract

Computational phenotyping has emerged as a practical solution to the incomplete collection of data on gender in electronic health records (EHRs). This approach relies on algorithms to infer a patient’s gender using the available data in their health record, such as diagnosis codes, medication histories, and information in clinical notes. Although intended to improve the visibility of trans and gender-expansive populations in EHR-based biomedical research, computational phenotyping raises significant methodological and ethical concerns related to the potential misuse of algorithm outputs. In this paper, we provide a narrative review of computational phenotyping of gender and examine its challenges through a critical lens. We also highlight existing recommendations for biomedical researchers and propose priorities for future work in this domain.

**Supplementary Information:**

The online version contains supplementary material available at 10.1186/s13293-025-00783-8.

## Highlights

- Sex and gender are inconsistently recorded in electronic health records (EHRs), limiting the scope of biomedical research using these data.
- Computational phenotyping algorithms attempt to fill these gaps by inferring gender-related information from patients’ historical health data.
- While these approaches aim to improve the visibility of trans and gender expansive people in biomedical research, they also introduce important methodological and ethical concerns, including (1) data quality issues, (2) underlying assumptions about gender, (3) bias in algorithm design and validation, and (4) potential for misuse.
- Future research should focus on building just and conceptually sound foundations for gender-based inquiry, such as creating and using measurement tools that accommodate fluidity, center lived experience rather than biological proxies, and allow for individualized data collection without defaulting to gender assignment.

**Supplementary Information:**

The online version contains supplementary material available at 10.1186/s13293-025-00783-8.

## Background

*“The light of big data creates big shadows.”* [1].

Data from electronic health records (EHRs) are foundational to biomedical research, underpinning studies across clinical medicine, public health, genomics, and health services research [2–6]. Sex and gender are widely recognized as critical variables for understanding health and illness and are often mandated for collection in EHRs [7–13]. EHR systems can capture sex and gender information across multiple fields with distinct clinical and operational meanings, including gender identity, sex assigned at birth, and legal or administrative sex. However, these fields, if available at all, are inconsistently populated across healthcare settings [14–20]. A study of 1.5 million adult patients at Mass General Brigham in the U.S. found that while legal sex was recorded for all patients, only 20% had information on gender identity or sex assigned at birth [21]. Similarly, in Ontario, Canada, just 0.8% of nearly 400,000 adult primary care patients had gender identity documented in their records [22]. At Rush University Medical Center in the U.S., only 25% of nearly 50,000 unplanned hospital admissions records included a populated gender identity field [16].

In response to these gaps, *computational phenotyping* has emerged as a means to augment incomplete data collection, particularly for gender-related information [23]. A computational phenotype is an algorithm that infers a patient’s gender based on information in their health record, such as diagnosis codes, medication codes for hormone prescriptions, procedure codes for gender-affirming care, and keywords in clinical notes [24–35]. This approach is a pragmatic solution to the limited uptake of sex and gender fields and has been used to collect data on trans and gender-expansive populations who have been historically underrepresented in EHR-based studies and biomedical research more broadly [36]. However, computational phenotyping brings significant methodological and ethical challenges that call into question both its validity and utility [37–40]. Given its potential to shape how gender is understood and operationalized within biomedical research, it is necessary to critically evaluate the data, assumptions, and design choices that underpin gender computational phenotypes.

As a diverse, interdisciplinary group of researchers, clinicians, and scholars who occupy social locations that position us at intersections of gender identity and expression, sexual orientation, race, and class, we respond to this need through a narrative review and critical evaluation of existing literature. Together, we draw on experiential knowledge shaped by both oppression and privilege, as well as professional and academic expertise across multiple theoretical and conceptual frameworks, to examine the methodological and ethical dimensions of computational phenotyping of gender. We identify four persistent issues across selected studies: (i) data quality, (ii) embedded assumptions about gender, (iii) bias in algorithm design and validation, and (iv) risks of misuse. Our analysis situates these issues within broader sociopolitical contexts to emphasize the importance of considering the environments in which computational phenotypes are developed and applied. We also outline existing recommendations for biomedical researchers and identify priorities for future research.

## Review of current practices

### Overview of computational phenotyping

**Fig. 1:**
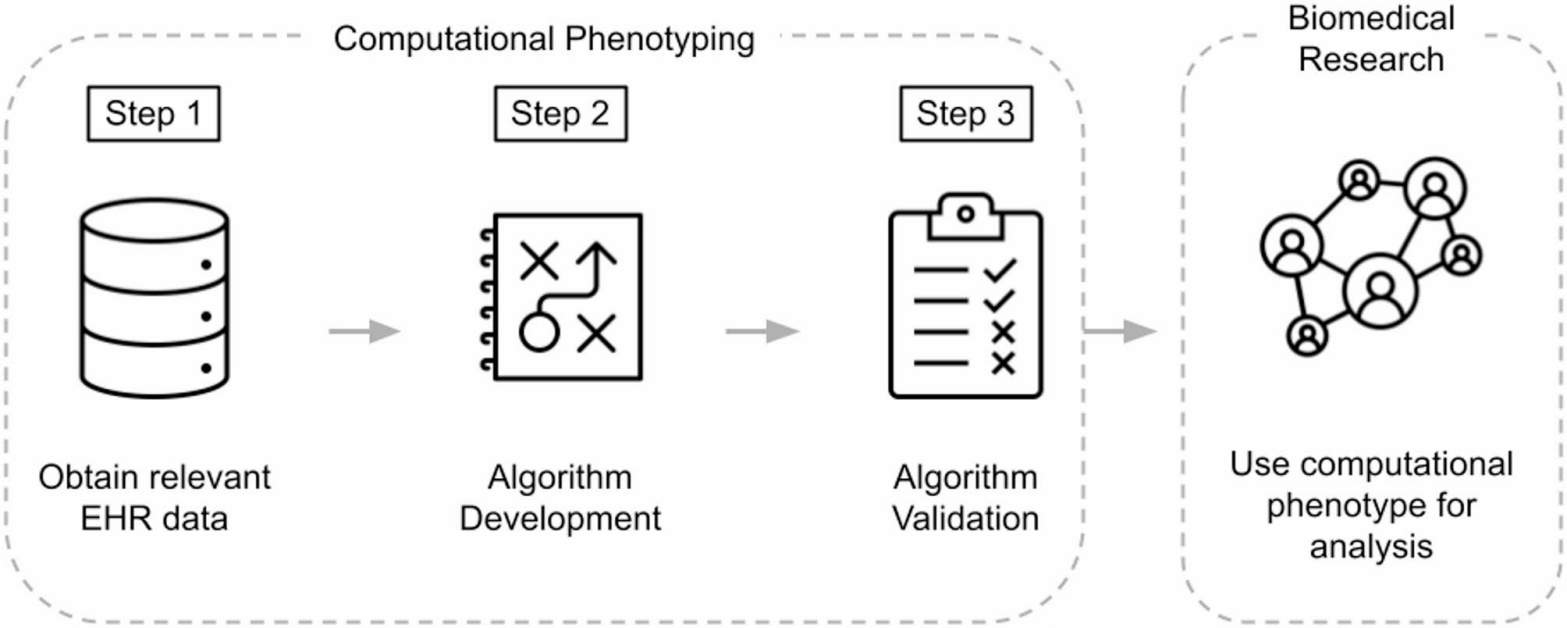
Overview of the computational phenotyping process. First, relevant structured (e.g., medical codes) and unstructured (e.g., clinical notes) data are obtained. Next, a rule-based or machine learning algorithm is used to develop the computational phenotype. Lastly, the accuracy of the algorithm is validated against a gold-standard label. The phenotype is then used in biomedical research to identify a study population or to derive an analytic variable

EHR-based biomedical studies rely on phenotyping, the process of identifying patients with particular characteristics or conditions (i.e., phenotypes) using data in their health records [41, 42]. Phenotypes are used to identify study populations as well as to extract variables for analysis [43]. When phenotype information is not explicitly available in a structured field (e.g., age or a lab test result), researchers develop a computational phenotyping algorithm to infer the phenotype based on a combination of structured and unstructured EHR data, such as diagnosis codes, medication prescriptions, and information in clinical notes (see Fig. 1).

Computational phenotypes can be developed through rule-based algorithms based on expert-derived criteria (e.g., a patient has the phenotype if their record contains relevant diagnosis and medication codes) or through machine learning models trained to predict phenotypes based on patterns in EHR data [42–44]. In either case, researchers validate the accuracy of the computational phenotype against a gold-standard label to ensure that it is suitable for downstream research. Gold standards are typically obtained through manual review of patient records, but may also be derived from lab test results or patient-reported data depending on the phenotype [45]. Accuracy is evaluated using standard performance metrics, including true and false positive rates and positive and negative predictive values [46–48]. Provided the computational phenotype is sufficiently accurate, it is then used as the basis for biomedical research. That is, it may be used to identify a study population (e.g., patients classified as having asthma by the algorithm for a study of treatment efficacy) and/or to derive variables of interest for analysis (e.g., a binary indicator of asthma status in a study of respiratory diseases).

### Computational phenotypes for gender

#### Motivation

While computational phenotyping has traditionally focused on common, chronic illnesses (e.g., asthma, heart failure), it has more recently been used to infer non-clinical characteristics due to incomplete or inconsistent documentation [43, 44, 49]. In the context of gender, existing algorithms primarily aim to identify trans^1^ and other gender expansive individuals in order to identify study populations for biomedical research studies [24, 51]. Historically, data on these populations is extremely limited due to structural oppression, including harassment and harm related to disclosure of identity, sparse research funding, and barriers faced by transgender researchers [52–54]. At the same time, existing studies show that transgender populations have disproportionately high rates of mental health distress, substance use, and HIV relative to cisgender populations [51]. Recognizing these disparities, several federal agencies have issued calls for action. A 2011 report from the Institute of Medicine (now the National Academy of Medicine) emphasized the need for more research at the intersection of LGBTQ + health and racial/ethnic minority health [55], and in 2016, the director of the National Institute on Minority Health and Health Disparities designated gender minorities as a “health disparity population for research purposes” [56]. EHRs, with their rich longitudinal and real-world data, offer a unique opportunity to study these populations at a scale and level of depth not possible in earlier research [28].

#### Existing literature

We identified 20 studies proposing gender computational phenotypes using a strategy following previous reviews [23, 43, 44]. The procedure used for study selection and article review is detailed in the Supplementary Materials. Here we summarize the data sources and methods used for algorithm development, the procedures for algorithm validation, and the study aims.

##### Data sources and methods for algorithm development

With the exception of the study by Hua et al., existing algorithms are rule-based, relying on combinations of medical codes, keywords, and/or sex and gender fields (see Table 1). Roblin et al. developed one of the first algorithms to identify transgender individuals at Kaiser Permanente Georgia based on diagnosis codes (e.g., codes related to sexual and gender disorders) and gender specific keywords in clinical notes (e.g., “transgender”, “transsexual”, “gender dysphoria”) [24]. Further information such as procedure codes was then used to discern female to male (FTM) and male to female (MTF) identity (e.g., codes for hysterectomy) [24]. This approach was later used by Quinn et al. to develop the Study of Transition, Outcomes, and Gender (STRONG) cohort using data from Kaiser Permanente Georgia and California [54]. The proposed algorithm consisted of three steps involving an initial identification of patients with at least one relevant diagnosis code or keyword followed by validation of transgender status and further stratification into transfeminine and transmasculine categories based on keywords, diagnosis codes, procedure codes (e.g., orchiectomy or hysterectomy), and use of hormone therapy (e.g., oestrogen or testosterone) [54]. Ehrenfeld et al. employed a similar approach within Vanderbilt University Medical System, identifying transgender people on the basis of having at least one relevant diagnosis code or keyword selected from previous literature, the authors’ expertise, and billing practices at the time [25]. Foer et al. introduced several transgender computational phenotypes based on diagnosis codes, keywords, gender identity fields, and discrepancies between gender identity, legal sex, and sex assigned at birth fields at Partners Healthcare (now Mass General Brigham) in Boston, Massachusetts [26]. Xie et al. also utilized diagnosis codes and keywords to identify individuals as “definitely”, “probably”, or “not” transgender using data from Kaiser Permanente Southern California [28].

**Table 1:** Overview of computational phenotyping algorithms for gender

Study	Data source	Gender label	Rule-based algorithm	Data used for algorithm development				
				Diagnosis codes	Medication codes	Procedure codes	Gender & sex fields	Clinical notes
Roblin et al. (2016) [24]	Kaiser Permanente Georgia	TG (MTF, FTM)	✔	✔		✔		✔
Quinn et al. (2017) [54]	Kaiser Permanente Georgia and Northern and Southern California	TG (TM, TF)	✔	✔	✔	✔	✔	✔
Ehrenfeld et al. (2019) [25]	Vanderbilt University Medical Center	TG	✔	✔				✔
Foer et al. (2020) [26]	Partners Healthcare	TG	✔	✔			✔	✔
Chyten-Brennan et al. (2020) [27]	Montefiore Health System	TGNB	✔	✔	✔		✔	✔
Xie et al. (2021) [28]	Kaiser Permanente Southern California	TG	✔	✔				✔
Alpert et al. (2021) [29]	CancerLinQ	TGNB	✔	✔			✔	
Guo et al. (2021) [30]	University of Florida Health	TGNC (TM, TF, unknown)	✔	✔	✔	✔	✔	✔
Wolfe et al. (2021) [31]	Veterans Health Administration	TG	✔	✔	✔		✔	
Dubin et al. (2022) [58]	NYU Langone Health	TG	✔	✔			✔	
Streed et al. (2023) [32]	Fenway Health	TGD (TGM, TGW)	✔	✔	✔	✔		
Hua et al. (2023) [33]	Mass General Brigham	TGD		✔		✔	✔	✔
Hines et al. (2023) [34]	University of Iowa Hospitals and Clinics	GE	✔	✔	✔		✔	
Nik-Ahd et al. (2023) [61]	Veterans Affairs Medical Centers	TG (TW)	✔	✔		✔		
Kim et al. (2024) [35]	Pediatric Emergency Department	TGNB	✔				✔	✔
Beach et al. (2024) [59]	Northwestern Medicine	TGNB	✔	✔			✔	✔
Ho et al. (2024) [57]	Utah-Based Healthcare System	TGD	✔	✔				
DeVone et al. (2025) [62]	Veterans Health Administration	TGD	✔	✔				
Engstrom et al. (2025) [60]	Mayo Clinic	TGD	✔				✔	
Symes et al. (2025) [63]	Tertiary Inner-City Emergency Department in Sydney, NSW, Australia	TGD	✔				✔	✔

TG = Transgender, MTF = Male to Female, FTM = Female to Male, TGNB = Transgender and Nonbinary, TGNC = Transgender and Gender-Nonconforming, TM = Transmasculine, TF = Transfeminine, TGD = Transgender and Gender Diverse, TGM = Transgender Men, TGW = Transgender Women, GE = Gender expansive

More recently, Chyten-Brennan et al. developed an algorithm to identify transgender and non-binary patients from Ryan White-funded clinics that provide dedicated HIV care within Montefiore Health System, the largest healthcare system in the Bronx neighborhood of New York City [27]. The algorithm supplemented diagnosis codes and keywords with gender-affirming medication prescriptions (e.g., concurrent male gender marker and estrogen prescription) and gender variables systematically reported to receive Ryan White HIV/AIDS Program funding (e.g., yes/no field for “transgender”) [27]. Within CancerLinQ, a database on people with cancer across practices within the U.S., Alpert et al. used diagnosis codes for gender identity disorder or transsexualism and variables derived from structured gender fields together with diagnosis codes (e.g., male gender and malignant neoplasm of the vulva) to identify transgender and nonbinary people [29]. At a Utah-based healthcare system, Ho et al. also used diagnosis codes to identify a cohort of transgender and gender diverse individuals [57].

Within academic medical centers, Guo et al. identified transgender and gender nonconforming people in the University of Florida Health Integrated Data Repository using a combination of diagnosis codes, keywords, medication prescriptions, demographic information, and procedure codes related to gender-affirming surgeries [30]. Dubin et al. relied on diagnosis codes and sex and gender fields to identify transgender patients at NYU Langone Health while Beach et al. also included clinical notes containing a term related to transgender or nonbinary identity within Northwestern Medicine’s data warehouse [58, 59]. At the University of Iowa Hospitals and Clinics, Hines et al. developed an algorithm to identify gender expansive individuals, including those who identify as transgender, nonbinary, transgender male or female, and other identities [34]. Their approach relied on discrepancies between legal sex, sex assigned at birth, and gender identity (excluding missing fields), as well as diagnosis codes for gender dysphoria or unspecified endocrine disorders and medication codes for estradiol or testosterone, which may indicate gender-affirming care [34]. Within the Mayo Clinic emergency department, Engstrom et al. identified transgender patients with a chief complaint of abdominal pain across four states (MN, WI, AZ, FL) using survey and registration data on sex assigned at birth and gender [60]. Similar to the study at the University of Iowa, patients were classified as transgender if there was a discrepancy between a sex and gender field or if the reported gender was non-binary [60].

Within the US Veterans Health Administration, Wolfe et al. used an analogous approach to identify transgender individuals based on diagnosis codes related to gender identity disorder and variables derived from codes for unspecified or not otherwise specified endocrine disorders, use of gender-affirming hormone therapy (i.e., hormones not associated with documented sex), and changes in the sex field [31]. Nik-Ahd et al. later built on this work to identify transgender women within the Veterans Affairs Medical Centers using diagnosis and procedure codes while DeVone et al. used only diagnosis codes to identify veterans with transgender and gender diverse identities [61, 62].

In contrast to the aforementioned studies that developed algorithms for entire populations of patients within a particular healthcare system, database, or institution, Streed et al. narrowed the scope of their study to evaluate the performance of a previously unvalidated algorithm for identifying transgender and gender diverse people with self-reported gender-identity data at Fenway Health, a Boston-based community health center specializing in care for sexual and gender minorities [32]. Similar to prior studies, the algorithm was based on the presence of transgender-related diagnosis and procedure codes as well as gender-affirming prescription data [32]. In another line of work, Kim et al. developed a computational phenotype to identify transgender and nonbinary individuals within a pediatric emergency department in the U.S. using keywords and gender and sex fields [35]. Symes et al. similarly developed a computational phenotype for trans and gender diverse people of all ages who presented to an inner-city emergency department in Sydney, New South Wales [63]. The authors utilized information in gender and sex fields, keywords related to a trans or gender diverse identity (e.g., “AMAB”, “NB_trans”, “MTF”), and whether a patients’ name included variations of “prefer”.

In recent years, machine learning methods have gained popularity as rule-based algorithms can be prohibitively resource-intensive to develop due to the complexity and variability of clinical documentation [44]. Only one study applied a machine learning approach to identify transgender and gender diverse patients within the Mass General Brigham healthcare system. Hua et al. first screened patients using sex and gender fields and medication prescriptions and then applied ClinicalBERT, a variation of bidirectional encoder representation from transformers (BERT) that has been pre-trained on biomedical text [33].

##### Algorithm validation procedures

To validate the performance of the computational phenotypes, gold-standard labels were most commonly derived from manual review of EHRs by trained annotators, a process referred to as “chart review” (see Table 2). This practice arises out of necessity as self-reported data is rarely fully documented in patient records [11, 14, 37]. For example, Beach et al. found that only 10% of their population had populated sexual orientation or gender identity fields [59]. Two notable exceptions are the studies of Streed et al. and DeVone et al., which relied on self-reported data for the gold standard [32, 62]. Streed et al. used self-reported data from Fenway Health that is collected at registration using a two-step method: first, by recording sex assigned at birth (male or female), and second, by documenting current gender identity (male, female, or another identity) [32]. DeVone et al. used self-reported data from 1.5 million veterans collected between 2019 and 2022 via VA.gov profiles or the VA’s 10-10EZ health benefits application form [62].

In terms of performance metrics, 11 of the 20 studies only evaluated the positive predictive value (PPV) of their final algorithm [24, 25, 27, 29, 31, 34, 54, 57, 59, 60, 63]. This is a relatively common practice within the phenotyping literature due to the time and expense of chart review [42–44, 46–48]. In these studies, the authors only assessed whether the algorithm correctly identified individuals within the category of interest (e.g., transgender or non-binary), without evaluating additional performance metrics on a random subsample of the full dataset. In contrast, 7 studies performed more complete validation [26, 28, 30, 32, 33, 61, 62] while 2 studies did not perform validation at all [35, 58].

Among the studies conducting complete validation, Foer et al. reviewed 324 randomly selected patient records and assessed the true positive rate (TPR), false positive rate (FPR), and PPV [26]. Nik-Ahd et al. reviewed five sets of 32 randomly selected charts (half with orchiectomy, half without) to evaluate five algorithms for identifying transgender women [61]. Guo et al. reviewed 100 charts and reported the TPR, FPR, PPV, negative predictive value (NPV), and F1-score [30]. Xie et al. considered the same performance metrics, but restricted their analysis to a random subset of 300 records containing relevant keywords (e.g., “transgender,” “transsexual”) [28]. Hua et al. also used a keyword list developed through expert input, existing literature, and a BioWordVec model to identify “potentially transgender individuals” and then reviewed 200 randomly selected records from this group for validation [33]. As Streed et al. evaluated performance of a previously developed algorithm, validation was performed on their entire sample of 52,746 individuals with self-reported data [32]. Similarly, DeVone et al. evaluated performance relative to the full 1.5 million all records with self-reported data within the Veterans Health Administration [62]. Generally, the algorithms were high performing, though we discuss limitations of the validation procedures in a subsequent section.

By comparison, Kim et al. did not perform a validation study and instead utilized chart review to iteratively develop their computational phenotype [35]. Dubin et al. also did not perform a validation study [58]. However, the authors reported that only 22% of records (334) in their identified cohort of transgender patients had both relevant diagnosis codes and patient or clinician-reported data indicating a transgender identity. The authors therefore suggested that a combination of these data must be used to fully capture gender minority populations.

**Table 2:** Accuracy of existing computational phenotypes

Study	Method for gold standard	Performance metrics				
		TPR	FPR	PPV	NPV	F1
Roblin et al. (2016) [24]	Chart review			0.68		
Quinn et al. (2017) [54]	Chart review			0.98		
Ehrenfeld et al. (2019)^† ^[25]	Chart review			0.97		
Foer et al. (2020) [26]	Chart review	1	0.73	0.08		
Chyten-Brennan et al. (2020) [27]	Chart review			0.84		
Xie et al. (2021) [28]	Chart review	0.97	0.05	0.95	0.97	0.96
Alpert et al. (2021) [29]	Chart review			0.76		
Guo et al. (2021) [30]	Chart review	1	1	1	1	1
Wolfe et al. (2021) [31]	Chart review			0.83		
Dubin et al. (2022) [58]	Not evaluated					
Streed et al. (2023) [32]	Self-reported gender and sex assigned at birth	0.87	0.01	0.89	0.99	
Hua et al. (2023)^†^[33]	Chart review	0.97	0.10	0.99	0.75	0.98
Hines et al. (2023) [34]	Chart review			1		
Nik-Ahd et al. (2023) [61]	Chart review	0.70	0			
Kim et al. (2024)^†^[35]	Chart review					
Beach et al. (2024) [59]	Chart review			0.70		
Ho et al. (2024) [57]	Chart review			1		
DeVone et al. (2025) [62]	Self-reported gender and sex assigned at birth	0.34	0	0.48	1	
Engstrom et al. (2025) [60]	Chart review			1		
Symes et al. (2025) [63]	Chart review			0.34		

Shown is the method used to obtain the gold-standard label and associated performance metrics for the final or highest performing algorithm. TPR = True positive Rate, FPR = False positive Rate, PPV = positive predictive Value, NPV = Negative predictive Value, F1 = F1 score

^†^Hua et al. used a machine learning model and also included results for accuracy, AUC, and AUPRC (0.96, 0.86, and 0.99, respectively). Kim et al. used the gold-standard to refine the keywords used in the algorithm and did not perform validation

##### Study aims

Of the 20 studies reviewed, 9 had the sole objective of developing and/or validating a gender computational phenotyping algorithm [26, 27, 30–33, 58, 59, 62]. The remaining studies had various secondary objectives. 4 studies aimed to estimate prevalence: Roblin et al. estimated the prevalence of transgender people within Kaiser Permanente Georgia [24], Alpert et al. estimated the prevalence of transgender and non-binary people within the CancerLinQ database [29], Hines et al. estimated the prevalence of gender expansive people within University of Iowa Hospitals and Clinics [34], and Nik-Ahd et al. estimated the prevalence of transgender people within the VA Medical Centers [61]. Another 4 studies focused on further characterizing the identified populations. Ehrenfield et al. evaluated healthcare utilization patterns among transgender people within Vanderbilt University Medical Center, finding that 50% had a diagnosed mental health condition, 14% were living with HIV, and 7% had diabetes [25]. Kim et al. characterized transgender and nonbinary patients within a prediatric emergency department presenting for psychiatric services, noting higher rates of repeat visits for high acuity psychiatric concerns and evaluations for suicidal ideation relative to patients outside of this population [35]. Similarly, Symes et al. characterized transgender and gender diverse patients at an emergency department in Sydney, Australia, identifying higher rates of hospital admission and high acuity presentations as well as increased use of mental health diagnostic codes [63]. Ho et al. evaluated the use of gender-affirming care among transgender and gender diverse individuals in a Utah-based healthcare system, reporting that over half of the population received gender-affirming hormone therapy and/or surgery [57]. 2 studies utilized their cohorts as part of clinical research. Xie et al. contributed to the multicenter Study of Transition Outcomes and Gender [28] while Quinn et al. used their method to develop a cohort for STRONG [54]. Lastly, Engstrom et al. had a secondary objective of matching transgender and gender diverse patients with cisgender patients with propensity score matching [60].

## Methodological and ethical issues

Despite the growing body of research proposing computational phenotypes, their development and application raise important methodological and ethical concerns. Much of the literature is thoughtful and self-critical, and we draw on many of the stated limitations within the selected studies to inform our analysis. Our review highlights four interrelated challenges: (i) data quality, (ii) embedded assumptions about gender, (iii) bias in algorithm design and validation, and (iv) risks of misuse. These issues are explored in detail in the following sections, supported by examples from the reviewed literature.

### Data quality

*“[B]efore there are data*,* there are people…”* [64].

**Fig. 2:**
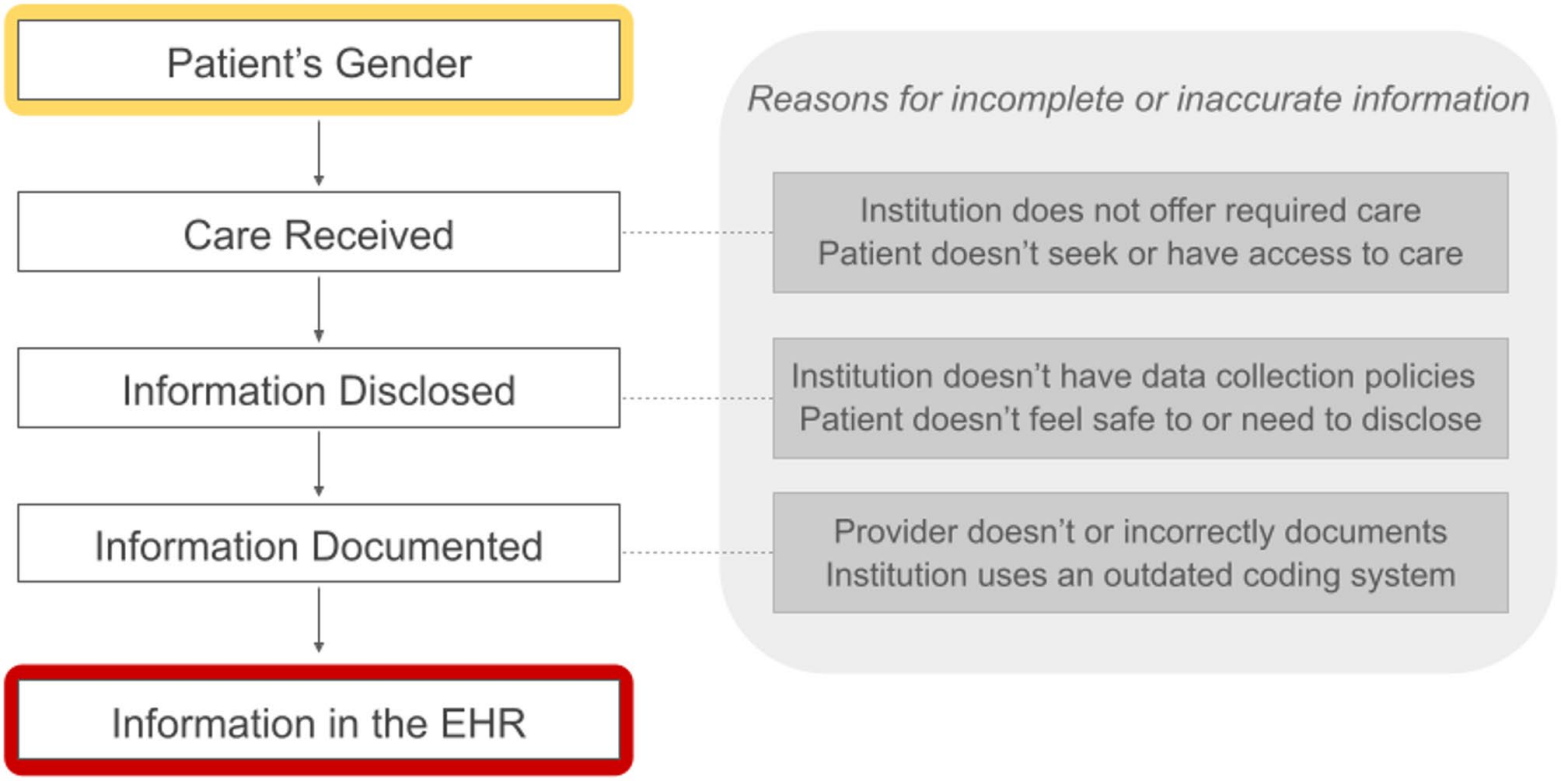
Process by which gender-related information enters an individual’s EHR and a non-exhaustive list of reasons for incomplete or inaccurate information

Information about gender recorded in EHRs is generally incomplete and inaccurate [65–68]. It is shaped by a complex interplay of factors, including the types of care individuals seek or are able to access, what they disclose during clinical encounters, and how healthcare institutions and providers document and interpret that information (see Fig. 2). As a result, the data do not reflect an individual’s self-identified gender, but rather how that identity is filtered through institutional practices and systemic bias [65, 69].

#### Systemic and institutional-level factors

At the institutional level, the collection of sex and gender data, if implemented at all, is often executed without reference to best practices [16, 19, 70]. It is frequently confined to specific clinical contexts such as psychiatry, endocrinology, or gender clinics, and carried out without adequate provider training [14]. For example, in developing a computational phenotype for transgender and nonbinary individuals with cancer, Alpert et al. found that most oncologists either do not ask about gender identity or do so in ways that make patients uncomfortable responding [29]. Beach et al. similarly note that when gender identity data is recorded by providers, rather than collected directly from patients, errors can arise from misinterpretation or personal bias [59].

Additionally, many of the data elements used for computational phenotyping, such as diagnosis and procedure codes, are derived from pathologizing and outdated documentation practices. For instance, “transgender” continues to appear on problem lists used to track current medical conditions and was a key component of an algorithm for identifying transgender patients within Partners Healthcare System [26]. Ehrenfeld et al. also discussed using diagnosis codes that contain outdated transgender-related terms, such as a code for “trans-sexualism with heterosexual history” [25], while Nik-Ahd et al. note that there is no training for clinicians on the use of diagnosis codes associated with “transgender” [61]. Moreover, data from problem lists and diagnosis codes do not represent individuals who do not pursue gender-affirming care, decline formal diagnosis, or who face structural barriers to accessing care, while also misrepresenting those who are documented in inaccurate or stigmatizing ways.

More generally, these issues illustrate the slow pace of institutional and coding reforms. It was not until 2019, with the release of the World Health Organization’s 11th edition of the International Classification of Diseases (ICD-11), that widely used diagnosis codes such as F64 (Gender identity disorders) and F65.1 (Fetishistic transvestism) were replaced by HA60 (Gender incongruence of adolescence or adulthood) [71]. This revision moved gender incongruence out of the mental disorders chapter and into one focused on sexual health, reflecting new “knowledge that trans-related and gender diverse identities are not conditions of mental ill-health, and that classifying them as such can cause enormous stigma” [58, 71]. However, adoption of ICD-11 has been uneven globally, partly due to the complexity of transitioning from ICD-10. For example, the United States has no firm timeline for ICD-11 implementation and took more than 20 years to complete the shift from ICD-9 to ICD-10 [72].

That said, even with widespread usage of more affirming coding standards, outdated codes will remain in patient records and continue to shape computational phenotypes. For example, an algorithm developed in one of the more recently published articles included in our review utilized ICD-10 code F65.1 (Fetishistic transvestism) to identify gender diverse veterans within the Veterans Health Administration [62]. While codes F64.0 (Transsexualism) and F64.1 (Gender identity disorder in adolescence and adulthood) were also included in the algorithm, F64.0 was only added to ICD-10 in 2017 when F64.1 was changed to “Dual role transvestism.” Since only the code label was changed, and not the code itself, many trans adults inappropriately have codes for “Dual role transvestism” in their EHRs [73]. This is evident in the study of Dubin et al., which explicitly utilizes F64.1 (Dual role transvestism) to identify transgender patients within NYU Langone Health [58].

Meanwhile, federal policies have changed what care institutions can offer, and in turn, what information is recorded in EHRs [74–76]. As part of a broader trend of banning gender-affirming care [77], the Veterans Health Administration has begun phasing out medical treatments for gender dysphoria in accordance with President Trump’s “Defending Women from Gender Ideology Extremism and Restoring Biological Truth to the Federal Government” executive order [78]. Consequently, diagnosis codes that often underpin computational phenotyping algorithms to identify transgender people, such as those developed using Veterans Health Administration data in the works of Wolfe et al., DeVone et al., and Nik-Ahd et al., will soon be erased [31, 61, 62].

#### Provider- and patient-level factors

At the provider level, documentation practices can reflect clinicians’ assumptions and personal understanding rather than patients’ self-identified gender. Ehrenfeld et al. noted that many providers misunderstood trans identities in their chart review process, finding ambiguous documentation of identities and pronouns used in non-affirming ways (e.g., “(s)he”) [25]. Guo et al. identified a similar phenomenon and referenced a clinical note wherein a provider misunderstood trans female, stating that a patient was “a male who is trans female (born female living as male) and currently taking testosterone cypionate for male hormone” [30]. Symes et al. found that misgendering and/or deadnaming occurred in 22.6% of discharge letters [63]. Misrepresentation or misunderstanding of a patient’s gender, whether intentional or unintentional, reflects a manifestation of structural bias that can delegitimize patients’ identities, contribute to clinical mistrust, and perpetuate inequities in care [79]. This bias also inevitably compromises the data for computational phenotyping by distorting the representation of gender in patient records and, in turn, leads to harmful misclassifications in algorithms’ outputs.

At the patient level, trans people disproportionately experience mistreatment in healthcare settings, with 24% of respondents in the 2022 U.S. Trans Survey reporting avoidance of care due to fears of being mistreated and another 24% not disclosing their gender to their healthcare providers [80]. Common negative experiences cited by patients include bias, discrimination, and disparaging comments from healthcare providers [12, 81, 82]. At the Veterans Health Administration, DeVone et al. found that over half of veterans with a relevant diagnosis code did not self-report a transgender or gender diverse identity, potentially due to concerns of stigma [62]. Beach et al. point to a similar issue in their study at Northwestern Medicine, where both patients and providers can enter gender identity related data into patient records [59]. When entered by the patient, the authors highlight that transgender individuals might intentionally align sex and gender fields either to affirm their identity or to avoid being classified as trans in their EHR. When entered by the provider, errors can occur due to misinterpretation, misunderstanding, or personal bias.

These experiences are further amplified by intersecting systems of oppression, including racism, sexism, ageism, and classism, that not only impact the care patients receive, but what information they disclose and how that information is documented in their EHR [83]. For example, Chyten-Brennan et al. found that their algorithm for identifying transgender and nonbinary people within HIV/AIDS clinics at Montefiore Health System was significantly less accurate for Hispanic people [27]. The authors suggest that disparities in data capture, particularly for immigrant and non-English-speaking communities, reflect broader systemic barriers to equitable care and documentation. Moreover, limited engagement and access to healthcare, especially for those at intersecting forms of marginalization, further contributes to the incomplete and inaccurate capture of gender information in EHRs. Chyten-Brennan et al. found that less than 1% of individuals were confirmed as transgender or nonbinary by their algorithm, which is significantly lower than anticipated [27]. The authors attribute this finding to stigma among HIV providers as well as disparate care engagement and disclosure among transgender and nonbinary people. Similarly, Nik-Ahd et al. found that 0.04% of the veteran population was confirmed as transgender, which is significantly lower than the estimated global prevalence. The authors state that this difference is likely due to the level of stigma that veterans have faced with political policies and the fact that clinicians may not be sufficiently or sensitively collecting data on gender [61].

### Assumptions about gender

*“Not everyone is male or female. Not everyone is cis or trans. The sooner we make space for these truths*,* the better.”* [84].

Trans people are those “whose gender or gender expression differs from expectations associated with the sex assigned to them at birth” [85]. As Os Keyes writes in *The Misgendering Machines*, this notion of difference encompasses a wide range of identities and experiences, including binary transitions, nonbinary or genderfluid identities, and people who don’t identify with any gender [86]. While gender theorists hold differing views on the nature of gender, they broadly agree that it is not “immutable, binary, or intrinsically linked to physiology” [86]. These insights challenge the core, though often implicit, assumption in phenotyping studies that gender is a fixed and essential trait that can be reliably extracted from historical data in a patient’s health records. This assumption is reflected in the use of oversimplified categorization schemes and in the ambiguous way gender is often operationalized in existing studies.

#### Categorization of gender

Most computational phenotypes adopt a binary classification scheme of “transgender” or “transgender or nonbinary” versus “not” [25–29, 31, 33–35, 57–60, 62, 63]. This model tacitly treats gender as static and singular, erasing its temporal and contextual variability and misrepresenting the lived experiences of many people. It also reinforces the false notion that gender must be stable to be measurable [87, 88]. Streed et al. and DeVone et al. explicitly acknowledged that a fundamental limitation of their algorithm is its inability to accommodate changes in gender over time [32, 62].

Seeking a more granular approach to gender classification, 5 studies further categorized individuals identified by their algorithm. Roblin et al. used a binary categorization of MTF and FTM [24], Quinn et al. stratified identified patients as either transmasculine or transfeminine [54], and Streed et al. classified individuals receiving hormone therapy as either transgender men or transgender women [32]. Nik-Ahd et al. focused specifically on identifying transgender women [61] and Guo et al. utilized three categories: transmasculine, transfeminine, and unknown [30]. While there have been considerable changes in terminology over the last several decades, with transmasculine and transfeminine becoming increasingly popular, categories aimed at identifying the “directionality of transness” can unnecessarily binarize nonbinary people and misportray those with additional genders (e.g., man, two-spirit) [14, 37]. Quinn et al. note that this is a key limitation of their algorithm and at the time of writing suggested that EHRs “alone are not sufficient for determination of non-binary gender identity” [54].

There is rich literature within sociology and informatics on best practices for categorizing and collecting data on gender [89–91]. The Williams Institute has developed two-step approaches for health surveys that first ask individuals if they identify within the binary and then follow with questions about transgender status [92]. Kronk et al. proposed a similar two-step collection process designed specifically for EHR systems, first inquiring about gender identity and then about the gender marker on an individual’s birth certificate [14]. When used to supplant self-report data, which is often regarded as the most accurate source of truth within EHR-based research [20, 93, 94], computational phenotyping models should be held to similar standards. However, phenotyping algorithms must inevitably work backwards from administrative or clinical indicators, such as diagnosis and procedure codes, that often reflect medical intervention rather than identity itself. This backward approach not only increases the risk of misclassification, but also reinforces a medicalized framing of gender and diminishes individuals’ agency in defining their own gender.

#### Operationalization of gender

The limitations surrounding the categorization of gender highlight the broader question of what computational phenotypes aim to measure. We have been deliberate in using the general term “gender” throughout our discussion^2^ as the output of phenotyping algorithms is often unclear and may reflect gender identity, expression, medical transition, or some combination thereof. Each of these concepts carries distinct implications for health and requires careful consideration when applied in biomedical research [95]. Gender identity refers to one’s internal sense of self and how one identifies, while gender expression involves the outward presentation of gender through appearance and behavior. Computational phenotypes, both in their design and in their validation, rely on proxies for these concepts, which are inherently imperfect and often overlap. For example, clinical notes may inconsistently or inaccurately capture gender expression or identity, self-reported information in sex and gender fields may conflate identity with administrative categories, and procedure codes related to medical transition capture only certain interventions and do not fully represent a person’s gender identity or experience. While many algorithms combine these data types to improve sensitivity, this approach comes at the cost of perpetuating an ambiguous operationalization of gender. Dubin et al. highlight this issue, noting that combining sex and gender fields with diagnosis codes conflates medical conditions with a dynamic identity category, as “diagnostic categories are not synonymous with the social identity categories captured by the demographic questions” [58].

### Algorithm design and validation

*“Algorithms are opinions embedded in code.”* [96].

The aforementioned challenges related to data quality, combined with assumptions about gender underlying computational phenotypes, introduce bias into both algorithm design and validation. These biases can foster overconfidence in an algorithm’s outputs and lead to flawed conclusions in EHR-based studies that rely on computational phenotypes.

#### Bias in design

Computational phenotypes are designed to capture individuals whose clinical encounters follow predictable and codifiable patterns. Many existing algorithms assume that gender can be inferred from clinical, biological, or administrative markers such as diagnosis codes, hormone prescriptions, and gender-affirming procedures. While this reliance on available EHR data is pragmatically necessary, it is also inherently reductive and pathologizing, as it encodes gender entirely within biomedical ontologies. For instance, diagnosis codes for gender dysphoria, transsexualism, or unspecified endocrine disorders often reflect reimbursement practices, medical necessity determinations, or outdated documentation standards. Many of these codes originate from historically pathologizing frameworks, including earlier versions of the ICD and Diagnostic and Statistical Manual of Mental Disorders (DSM), which classified gender diversity as a form of mental illness or sexual deviance [71]. As a result, computational phenotypes tend to capture only individuals whose gender-related care is both medicalized and well-documented [24, 25, 30, 31]. Those who do not disclose their gender, follow non-normative care pathways, or who avoid or are unable to access gender-affirming care are likely underrepresented. Beach et al. note that many trans and nonbinary patients are never formally diagnosed with gender dysphoria, and that using a diagnosis code can unnecessarily medicalize gender identity as a disease state, potentially increasing stigma [59]. Ho et al. highlight that some people who experience gender dysphoria or gender incongruence may not disclose this information to clinicians due to mistrust of the healthcare system or request that related diagnosis codes not be used due to insurance coverage or use of parental insurance [57]. Notably, Alpert et al. found that their algorithm based on diagnosis codes and sex and gender fields “would have identified 0.003% of patients seen at CancerLinQ practices as of October 2019 as transgender,” which is a substantial underestimate [29]. This exclusion can in turn distort downstream analyses, for example, by leading to conclusions that trans people are more likely to be white or concentrated in the Northeastern and Western areas of the U.S [37].

Hines et al. explicitly note that reliance on proxies for medical intervention excludes a “substantial portion of gender-diverse populations” [34] and suggest that incorporating self-reported sex and gender fields can lead to more representative computational phenotypes. However, self-reported data, if available at all, are constrained by the limited response options permitted within most EHR systems. In an analysis from the University of Iowa Hospitals and Clinics, the same authors found that adolescent and young adult populations often report identities that fall outside of these predefined categories (e.g., agender, demiboy, genderqueer, transfeminine) [34]. Beach et al. similarly observed that preprogrammed categories with EHR systems are limited [59]. In their study, a specific option for “nonbinary” was not available [59]. As a result, participants who did not identify as female, male, transgender male, transgender female, or who did not choose “prefer not to disclose” or “unknown,” were required to select the “other” category, which can be both stigmatizing and isolating [59]. Kim et al. proposed expanding gender identity fields to include terms such as “nonbinary,” “gender fluid,” and “unsure/questioning,” which frequently appear in free-text entries within patient records [35].

In response to these limitations, researchers often rely on constructed variables that indicate discrepancies between sex and gender fields (e.g., gender identity recorded as ‘male’ and sex as ‘female’ [27]) or patterns in prescription data (e.g., male gender marker and estrogens/progestins, estrogen, or progesterone and spironolactone 200 mg [30]). While these efforts aim to better capture gender diversity within the constrained structure of EHR data, they embed assumptions about bodies and medical transition pathways, and fail to account for identities that are nonbinary, fluid, neutral, or evolving over time [59]. Moreover, Foer et al. and Nik-Ahd et al. reported that this approach was particularly inaccurate [26, 61]. At Partners Healthcare in Boston, all patients flagged based solely on discrepancies across sex assigned at birth, legal sex, and gender identity fields were ultimately found to be cisgender upon chart review [26]. Within the Veterans Health Administration, relying solely on sex and gender related fields resulted in an algorithm with 13% accuracy [61]. DeVone et al. also noted that individuals with mismatched sex and gender fields may represent distinct subgroups [62]. For instance, they may differ from those who explicitly identify as nonbinary in their self-reported data.

While many studies turned to keywords within clinical notes to address the limitations of structured data, keyword selection generally mirrors prevailing clinical documentation practices, rather than reflecting current or culturally relevant language, particularly for people of color and nonbinary individuals. For example, the computational phenotype developed by Roblin et al. relied on a very narrow set of keywords, including “transgender,” “transsexual,” “transvestite,” “gender,” “gender dysphoria,” and “gender reassignment” [24]. Although subsequent studies have broadened their keyword sets, it remains impossible to fully capture the diversity of gender terminology, let alone to assume that this diversity is adequately reflected in EHR data. Ehrenfeld et al. speculated that adding more keywords would improve the accuracy of their algorithm (e.g., “nonbinary”, “genderqueer”), but pointed out that language is bound to change and that identities will need to be continuously added [25]. Similarly, Xie et al. emphasized that keyword lists will require ongoing revision to remain current with evolving language [28]. In an effort to move beyond keyword-based methods, Hua et al. utilized a deep learning approach to identify gender-diverse individuals without relying on manually selected terms [33]. However, their model struggled with contextual understanding, for example confusing terms like “hysterectomy” and “they/them”, and was trained primarily on PubMed and social media posts due to limited access to large-scale EHR data [97]. These issues underscore the limitations of both rule-based and machine learning algorithms when applied to contexts where data on gender are incompletely or inaccurately documented.

#### Bias in validation

Bias in algorithm design is further compounded by flawed validation practices. Most studies utilize chart review to obtain the gold-standard label, which rests on the assumption that annotators can accurately infer a phenotype from a patient’s historical EHR data. While this assumption may be reasonable for well-documented chronic conditions that have traditionally been the focus of computational phenotyping (e.g., asthma, heart failure), it is unlikely to hold true for gender [23, 24]. Much like the outputs of the algorithms themselves, it is unclear whether the gold-standard is meant to reflect gender identity, expression, medical transition, or some combination of these factors. For instance, chart review often only identifies a patient as transgender if there is explicit documentation of gender dysphoria or evidence of medical transition. In one study, Alpert et al. limited their review to records containing relevant diagnosis codes, leading to the misclassification of some transgender patients as “not transgender” [29]. More broadly, the absence of documentation is not neutral [98]. It may reflect erasure, patient mistrust, or systemic failures to solicit or record information.

These limitations have important implications for the use of computational phenotypes in biomedical research. When algorithms are evaluated against such flawed reference standards, even high accuracy metrics are misleading (see Table 2). As an illustrative example, consider a simple analysis aimed at estimating the prevalence of a clinical condition among individuals identified as transgender by a computational phenotype. Even if the algorithm appears perfectly accurate relative to a chart-reviewed gold standard, its utility is compromised if the gold standard itself has low sensitivity. In such cases, many individuals will be excluded from both the gold standard and the algorithm’s outputs, leading to biased estimation of the disease prevalence and a distorted understanding of the condition’s impact on trans populations. This example highlights how limiting biomedical research to individuals who are legible to algorithms can perpetuate incomplete or skewed representations of transgender health and, in doing so, obscure the very populations that computational phenotypes intend to make visible.

Unfortunately, this concern is not hypothetical. In a related study, Manfredi et al. used insurance claims data to examine cancer outcomes among transgender women, reporting a lower prevalence of prostate cancer among those receiving gender-affirming hormone therapy, but a positive correlation between hormone use and aggressive disease [99]. In response, Hamnvik et al. and Berner et al. raised methodological concerns in two separate letters to the editor [38, 39]. Foremost among them was the use of diagnosis codes to identify transgender women. These codes are known to have low sensitivity and may also capture individuals undergoing treatment for other conditions, such as orchiectomy or prostate cancer, resulting in substantial misclassification. The study’s finding that only 31.5% of transgender women had records of hormone therapy, compared to 71% in similar datasets, further underscores this concern [99]. As both sets of authors argue, such misclassification not only compromises the validity of the study’s findings, but also risks reinforcing harmful narratives that could jeopardize access to hormone therapy for transgender women.

### Potential for misuse

*“[S]urveillance is a central practice through which the category of transgender is produced*,* regulated*,* and contested.”* [100].

In addition to methodological challenges, computational phenotyping raises significant ethical concerns, particularly regarding the potential misuse of algorithmic outputs. Although not consistently addressed across studies, Chyten-Brennan et al. highlight the risk of identifying transgender and nonbinary patients in environments of pervasive discrimination [27]. Reflecting these concerns, Hua et al. excluded individuals who chose not to disclose information in sex and gender fields when developing their phenotyping algorithm [33]. Beach et al. emphasize that this is a necessary practice in order to ensure that patient privacy and autonomy are prioritized and that gender identity data should never be obtained without explicit consent [59]. More broadly, Hines et al. point out the dangers of recording gender identity in EHRs, citing risks such as bias, discrimination, and mistreatment by healthcare providers [34]. These risks are especially pertinent for children and adolescents, whose medical information is accessible to parents or guardians [82].

In related work, Alpert et al. examine the principle of beneficence in transgender health research using insurance claims data [37]. They point out that even de-identified datasets carry a “theoretical, but plausible” risk of reidentification, particularly within small populations [101, 102]. Critically, the authors also highlight that computational phenotyping can involve identifying individuals receiving gender-affirming care without an explicit diagnosis, such as those with a code for an unspecified endocrine disorder or relevant keywords in clinical notes [37]. In the current political climate marked by the criminalization of gender-affirming care [103], attempts to access transgender patients’ health records [104], and systemic efforts to erase trans identities, computational phenotyping can become a tool of surveillance that amplifies discrimination, misclassification, and inflicts harm [105, 106].

Similar warnings have been made in the context of automated gender recognition tools, such as those used in airport body scanners [100]. Scholars of technology and ethics have broadly critiqued such “processes of technologization and rationalization that frame bodies, identities, and groups as outside of historical frameworks and experiences of racial and other forms of difference” [107]. Likewise, computational phenotyping, when abstracted away from its broader sociopolitical context, risks reinforcing the structural conditions it purports to address. At this moment, when the stakes are not merely theoretical, interdisciplinary researchers and scholars must seriously evaluate potential benefits of algorithmic development against the substantial and potentially life-threatening risks to already vulnerable populations [64, 108]. This is particularly evident in ongoing debates over algorithmic race classification, which offer several important lessons for computational phenotyping.

#### Lessons from algorithmic race classification

Within healthcare and biomedical research, algorithmic race classification is the automated and predictive assignment of race from proxies like demographics and clinical data, often without self-identification or consent [109, 110]. For example, Gichoya et al. found that deep learning models can infer self-reported race from medical imaging data alone, even when images are cropped, corrupted, or noised, and with performance generalizing across imaging modalities and healthcare settings [110]. This finding poses an enormous risk, as such models are a direct vessel for the reproduction and exacerbation of race-based disparities that exist within healthcare. The danger is further compounded by the fact that human oversight is of limited use to recognize and mitigate these race-based disparities as clinicians cannot accurately identify racial identity from medical images themselves. As the authors warn, “if an AI model relies on its ability to detect racial identity to make medical decisions, but in doing so produced race-specific errors, clinical radiologists (who do not typically have access to racial demographic information) would not be able to tell, potentially leading to errors in health-care decision processes” [110].

Critical scholars caution against algorithmic race classification altogether on the premise that the practice risks reinscribing race as a biological concept, which is an outdated pseudoscientific claim that has historically been used to rationalize slavery, eugenics, and other social inequalities [86, 111–114]. Other researchers point out that even self-identified or self-reported race does not necessarily align with biological traits, further highlighting the conceptual contradictions underlying algorithmic race classification [115]. While Gichoya et al. acknowledge that race is a social rather than a biological construct, and that more genetic variation exists within racial groups than between them, they also maintain that self-reported race remains a strong proxy for racial identity [110]. This claim risks reifying race as a biological construct or causal factor rather than a fluid social construct. Many critical scholars instead situate algorithmic race classification in a political context, shaped by colonial logics of surveillance, governance, and control [112, 116, 117]. From this perspective, race-classifying algorithms do not merely reflect social categories, they reproduce and automate racial hierarchies under the guise of neutrality and objectivity, perpetuating racist science and deepening the marginalization of racialized communities [86, 111].

Similarly, computational phenotyping rests on the premise that gender can be predicted from proxies rather than attempting to capture gender as social, fluid, and self-determined [23]. In both cases, identity is rendered visible through logics of surveillance, which omits the opportunity to disclose identity through individual agency. Applied to gender, this surveillance logic further extends what Ruha Benjamin calls the “New Jim Code,” which refers to the coded and automated reinforcement of inequities through technical systems that are deeply embedded with racialized and gendered assumptions, but are made to appear neutral or objective [111]. As Os Keyes argues in *The Misgendering Machines*, algorithmic systems that attempt to classify gender often make ontological claims about what gender is, reducing it to a binary and ignoring its fluid and socially constructed nature, thereby excluding and harming those who identify as non-binary [86]. These systems also presume that gender is physiologically rooted, essentializing the body as the source of truth, further harming and discriminating against those who identify as trans. These issues strongly parallel the problems with algorithmic race classification, where harm exists not only in the errors made by the algorithms, but also with proxy-based and data-driven construction of identity, which reproduces categories that historically and contemptuously pathologize racialized and gendered individuals in ways that are bound by outdated and harmful colonial logics. Rather than reform these practices through community-based inclusivity or more representational data sets, many critical scholars across Black studies, data justice studies, and trans studies urge us to challenge the legitimacy and necessity of algorithmic classification itself, especially considering the historical and political contexts in which it is situated and the potential harms that are at risk of being produced [86, 111, 112, 116, 117].

## Conclusions

*“When approaching any new source of knowledge…it’s essential to ask questions about the social*,* cultural*,* historical*,* institutional*,* and material conditions under which that knowledge was produced…”* [64].

While computational phenotyping of gender is increasingly used in EHR-based biomedical research, it raises significant methodological and ethical concerns that challenge the validity, and ultimately the utility, of this practice. Phenotyping attempts to infer gender through clinical, biological, or administrative proxies, which is methodologically flawed and conceptually problematic. It also risks perpetuating a history of using gender in ways that have contributed to scientific misrepresentation and social injustice, making even well-intentioned studies susceptible to causing harm. We close by outlining existing recommendations for biomedical researchers and identifying priorities for future research.

### Existing recommendations

In related work examining the ethics of identifying and researching transgender and gender-diverse individuals from insurance claims data, Alpert et al. apply the framework of epistemic justice to propose six recommendations for minimizing harm and maximizing benefits for transgender individuals and communities (see Table 3) [37]. Generally, the reviewed studies did not follow all of these principles. For example, only four of the selected studies included a positionality statement, one identifying the lead author as a cisgender male [35], one identifying stakeholder involvement from transgender patients [54], and two identifying LGBTQ + community members or allies as authors or involved in study oversight [57, 59]. Cato et al. invoke the Belmont Report’s principles of respect for persons, beneficence, and justice to highlight a range of ethical issues in EHR phenotyping more broadly [118]. These issues include patient consent for secondary data use, the balance of harms and benefits in research based on phenotyping, and the influence of clinician bias, whether conscious or unconscious, on study design and findings. The authors advocate for greater community consultation, transparency in data use, privacy-preserving approaches, and dynamic or portable consent models that return more control to patients. Comparable calls have emerged outside the EHR phenotyping literature [119]. For example, building on decades of scholarship on how science can serve marginalized populations, Kennis et al. propose four concrete actions for researchers: establishing advisory boards with transgender representation, assembling multidisciplinary teams, prioritizing life-saving research, and restructuring the ethical approval process [120]. While these recommendations are valuable and actionable, we build on them by taking a complementary, though more critical, stance informed by recent scholarship conceptualizing how gender is understood and operationalized in scientific research.

**Table 3:** Suggestions from Alpert et al. [37] to minimize harm and maximize benefit to transgender individuals and communities when using insurance claims data for biomedical research

1. Explicitly describe the categories that are utilized (e.g., people with cervixes as evidenced by procedure codes)
2. Explicitly acknowledge data limitations and dangers to transgender communities
3. Use reflexivity by which researchers state their positionality and biases to contextualize their work
4. Prevent identifiability of transgender individuals
5. Transgender researchers—especially those with multiple marginalized identities—lead or co-lead research, guide analyses, and interrogate the work’s ethics and utility
6. Supplement claims-based research conducted on transgender people with community-based participatory research conducted by and alongside transgender people

### Priorities for future research

In a 2024 *Nature* special collection exploring the risks and challenges of integrating of sex and gender into research, Ashley et al. highlight the inadequacy of current terminology for gender-based research, arguing that it lacks both the pragmatism required for scientific inquiry and the flexibility needed to reflect the diversity of human experience [84]. To address this gap, the authors introduce the concept of *gender modality* [121], defined as the relationship between a person’s gender identity and the gender assigned at birth. Much like the concept of sexual orientation which has moved us away from a gay/straight binary, this framework includes familiar categories like cisgender and transgender, while also capturing a broader range of experiences (see Table 4). Gender modality is a concept that is already in use by transgender communities, clinicians, and policymakers, and has been applied by Statistics Canada, Planned Parenthood, and the Supreme Court of Canada [84]. Ashley et al. argue that this shift in terminology can improve scientific inquiry in three ways: (1) expanding how gender is categorized and captured in data, (2) refining research questions and interpretations, and (3) forcing greater clarity on what investigators are actually measuring. While no single framework can resolve all challenges, establishing more nuanced language is an essential step toward biomedical research that embraces, rather than simplifies, the complexity of gender.

**Table 4:** A non-exhaustive list of gender modalities provided in Ashley et al. [84]

Modality	Definition
Agender	People who do not identify with any gender
Cisgender	People whose gender identity corresponds to the gender they were assigned at birth
Closeted trans people	Individuals whose gender identity does not correspond to the gender they were assigned at birth, but who do not share their gender identity publicly
Culture-specific identities	Individuals can have identities, such as Two-Spirit identities in North American Indigenous communities and hijra on the Indian subcontinent, that might not align with Western concepts of gender and sexuality. People with these identities might not consider themselves cis or trans because of the Western philosophies that underpin these terms
Detrans/retrans	People who have ceased, shifted or reversed their gender transition
Gender questioning	People who are unsure of their gender identity and are in the process of working it out
Intersex	People who were born or who endogenously developed sexual traits that differ from typical expectations of female and male bodies. Some intersex people do not consider themselves to be cis or trans
People with dissociative identity disorder whose alters have distinct gender identities	People with this condition, also known as plural people, can have several identities, known as alters or headmates, that have distinct gender identities. These alters can have different gender modalities
Raised in a gender-neutral manner	People who were raised without being referred to as a boy/he or girl/she until they were old enough to express their gender identity
Transgender	People whose gender identity does not correspond to the gender they were assigned at birth

The lack of adequate terminology underscores a more fundamental problem: gender has yet to be fully conceptualized within scientific practice, and it is unclear whether a complete conceptualization is currently possible or desirable. As noted by Restar et al. in the context of epidemiological studies, “[M]easuring gender and sex has no gold standard, perhaps since these variables depend on time and context. Pragmatically, research questions, aims, scope, study design, methods, and capacity to collect and analyze data should all influence how to measure gender and sex. That is, as there is no single best practice, investigators must decide which dimensions of sex and gender are relevant to their research questions.” Beach et al. note that it is also critical to “refine, study, and further standardize collection of sexual orientation and gender identity data in a manner which centers patients, respects autonomy and privacy, and clearly facilitates justice to LGBTQ+ people” [59]. Numerous scholars have also challenged the validity of gender variables in science more broadly [86, 111, 112], arguing that existing categories are embedded in histories of pseudoscience and structural injustice. Therefore, measurement can never be “fixed” within existing systems, but instead must be reimagined or abandoned entirely. Within EHR-based research, rather than attempting to reverse-engineer gender with computational phenotypes from distorted and incomplete data shaped by a legacy of structural oppression, we argue that researchers should shift their focus toward developing a just and conceptually sound foundation for gender-based research.

Practically, this means creating and using measurement tools that accommodate fluidity, center lived experience rather than biological proxies, and allow for individualized data collection without defaulting to gender assignment. This echoes efforts like Kronk et al.’s framework for transgender data collection in EHR systems [14], as well as rethinking gender measurement more broadly through the lenses of data justice and intersectional feminist and queer theory [64, 122]. With access to larger and larger health data sets, researchers and scholars must collectively welcome and critically engage with questions that appear deceptively simple, such as: What exactly are we trying to measure? Can it be measured? How has it been measured before, and who might be harmed by these approaches? Without grounding scientific work in ethical frameworks, sociopolitical context, and epistemic reflexivity, we risk perpetuating the very structures of marginalization that we seek to challenge [88]. 

## Supplementary Information

Supplementary Material 1.

## Data Availability

No datasets were used or analyzed for this study.

## Abbreviations

EHR: Electronic health record
Trans, TG: Transgender
MTF: Male to female
FTM: Female to male
TGNB: Transgender and nonbinary
TGNC: Transgender and gender-nonconforming
TM: Transmasculine
TF: Transfeminine
TGD: Transgender and gender diverse
TGM: Transgender men
TGW: Transgender women
GE: Gender expansive

## Footnotes

1 See Table 4 for definitions of gender modalities. Occasionally transgender is shortened to trans [50].

2 However, when referencing publishing articles, we adhere to their original terminology.
