# Supplementary material for: When algorithms infer gender: revisiting computational phenotyping with electronic health records data
Source: Biol Sex Differ. 2025 Dec 31;17:16. doi: 10.1186/s13293-025-00783-8 (PMC12865949; doi:10.1186/s13293-025-00783-8)
Supplement: Supplementary file 1 — Supplementary Material 1. [file 13293_2025_783_MOESM1_ESM.pdf]

Supplementary Materials

Literature search procedure

We conducted our literature review using a strategy based on a previous narrative review that focused more broadly on computational phenotyping within electronic healthcare data (e.g., EHRs, claims, and other administrative data) to identify transgender people [23] and two previous reviews that focused on the general problem of phenotyping with EHRs [43,44]. It is important to note that our goal was to conduct a narrative, rather than systematic, review of the literature on computational phenotyping of gender. We include our search methodology for transparency and in an effort to minimize bias as much as possible [123].

Briefly, we searched PubMed and Web of Science for articles that focused on phenotyping gender with EHR data through September 29, 2025. The detailed search queries are provided in **Table S1**. After removing duplicates, articles were retrieved and underwent title and abstract screening by one author (L.D.). Articles were excluded if they (i) did not focus on gender computational phenotyping, (ii) did not use EHR data, or (iii) were conference abstracts or review papers.

Two authors (L.D. and D.S.C) reviewed the full-text articles and another author (J.G.) verified the information from the full-text review. After excluding papers that did not focus on gender computational phenotyping with EHR data, 20 papers were selected (**Figure S1**). During the full-text review, we extracted information on: (i) the data sources used, (ii) the phenotype(s) considered, (iii) the methods applied for algorithm development, (iv) the algorithm validation procedures, (v) the study aims, and (vi) the stated limitations and ethics considerations.

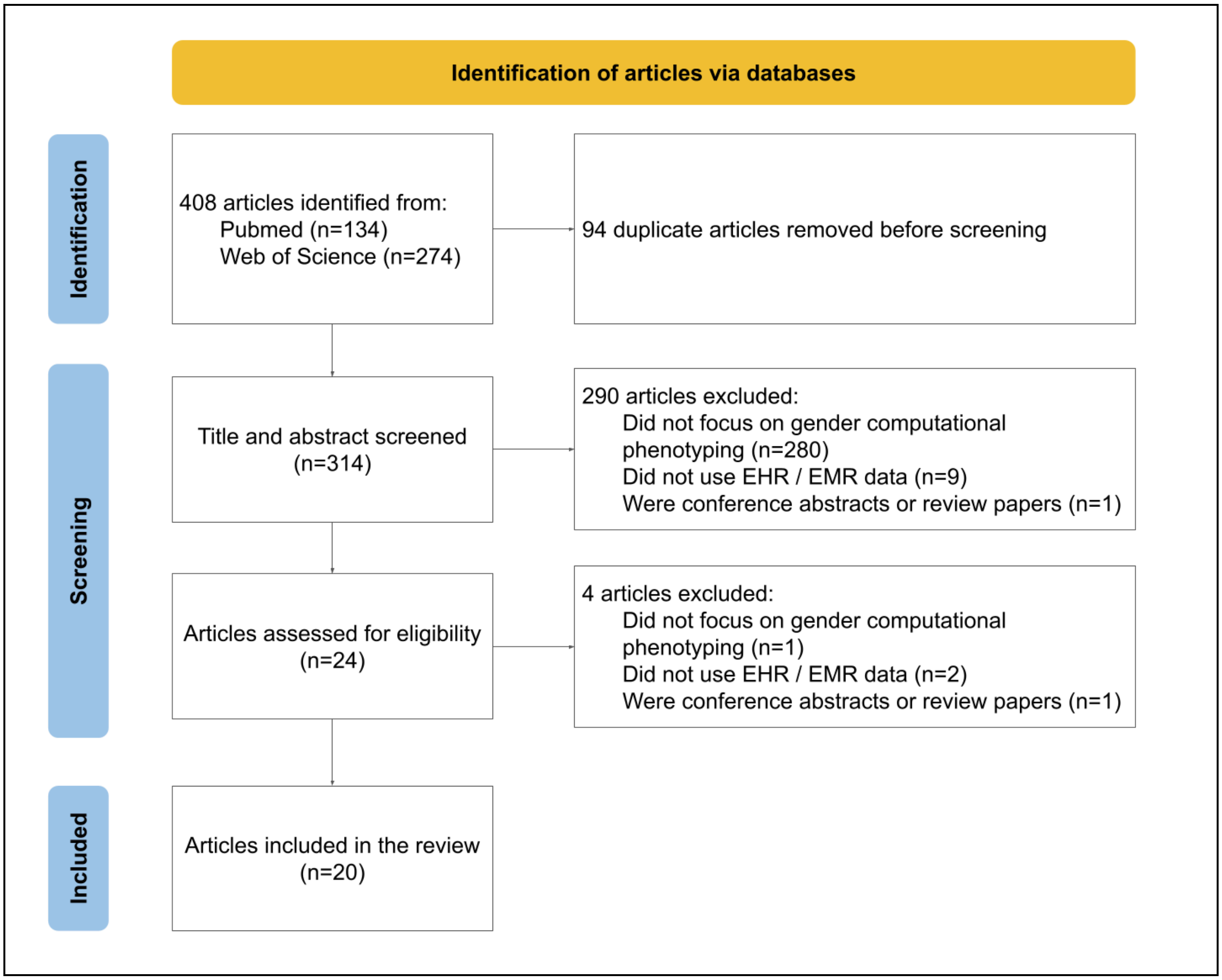

Figure S1. Flow diagram of articles included in the narrative review.

Table S1. Database search strategy.

**Database**	**Search String**
<u>PubMed</u><br>(n = 134)	("electronic health records" OR "electronic medical records" OR "EHR" OR "EMR" OR "EHRs" OR "EMRs" OR electronic health records[Mesh] OR electronic medical records[Mesh] OR "electronic health record"[tiab] OR "electronic medical record"[tiab] OR "health record*"[tiab] OR "health data*"[tiab] OR "administrative data"[tiab]) AND (computation* OR computer* OR phenotyp* OR "phenotype"[Mesh] OR algorithm* OR identifying[tiab] OR identification[tiab]) AND (transgender*[tiab] OR "Transgender Persons"[Mesh] OR "Health Services for Transgender Persons"[Mesh] OR "gender nonconform*"[tiab] OR transmen[tiab] OR "trans men"[tiab] OR transwomen[tiab] OR "trans women"[tiab] OR TGNC[tiab] OR TGNB[tiab] OR "gender identity"[tiab] OR "gender minorit*"[tiab] OR "gender identification"[tiab] OR "gender-affirming"[tiab] OR genderaffirming[tiab] OR "gender affirming"[tiab] OR "transgender-related"[tiab] OR "gender diverse"[tiab] OR "gender-diverse"[tiab] OR "gender-affirming"[tiab] OR "gender identity"[tiab] OR "gender-identity"[tiab] OR "gender minority"[tiab] OR "gender minorities" OR "gender clinic"[tiab] OR "transmasculine"[tiab] OR "transfeminine"[tiab] OR "gender dysphoria"[tiab] OR "gender identity disorder"[tiab] OR "transgendered"[tiab] OR non-disclosed [tiab])
<u>Web of Science</u><br>(n = 274)	S=("electronic health records" OR "electronic medical records" OR EHR OR EMR OR EHRs OR EMRs OR "electronic health record" OR "electronic medical record" OR "health record*" OR "health data*" OR "administrative data")<br>AND<br>TS=(computation* OR computer* OR phenotyp* OR algorithm* OR identifying OR identification)<br>AND<br>TS=(transgender* OR "Transgender Persons" OR "Health Services for Transgender Persons" OR "gender nonconform*" OR transmen OR "trans men" OR transwomen OR "trans women" OR TGNC OR TGNB OR "gender identity" OR "gender minorit*" OR "gender identification" OR "gender-affirming" OR genderaffirming OR "gender affirming" OR "transgender-related" OR "gender diverse" OR "gender-diverse" OR "gender identity" OR "gender-identity" OR "gender minority" OR "gender minorities" OR "gender clinic" OR transmasculine OR transfeminine OR "gender dysphoria" OR "gender identity disorder" OR transgendered OR "non-disclosed")<br><br>**Search in** - All Databases<br>**Collections** - All
